# Weakly Supervised Sensitive Heatmap framework to classify and localize diabetic retinopathy lesions

**DOI:** 10.1038/s41598-021-02834-7

**Published:** 2021-12-08

**Authors:** Mohammed Al-Mukhtar, Ameer Hussein Morad, Mustafa Albadri, MD Samiul Islam

**Affiliations:** 1 Computer Center, University of Baghdad, Baghdad, Iraq; 2 Al Khwarizmi College of Engineering, University of Baghdad, Baghdad, Iraq; 3 Department of Computing Science, University of Alberta, Edmonton, Canada

**Keywords:** Biomedical engineering, Electrical and electronic engineering

## Abstract

Vision loss happens due to diabetic retinopathy (DR) in severe stages. Thus, an automatic detection method applied to diagnose DR in an earlier phase may help medical doctors to make better decisions. DR is considered one of the main risks, leading to blindness. Computer-Aided Diagnosis systems play an essential role in detecting features in fundus images. Fundus images may include blood vessels, exudates, micro-aneurysm, hemorrhages, and neovascularization. In this paper, our model combines automatic detection for the diabetic retinopathy classification with localization methods depending on weakly-supervised learning. The model has four stages; in stage one, various preprocessing techniques are applied to smooth the data set. In stage two, the network had gotten deeply to the optic disk segment for eliminating any exudate's false prediction because the exudates had the same color pixel as the optic disk. In stage three, the network is fed through training data to classify each label. Finally, the layers of the convolution neural network are re-edited, and used to localize the impact of DR on the patient's eye. The framework tackles the matching technique between two essential concepts where the classification problem depends on the supervised learning method. While the localization problem was obtained by the weakly supervised method. An additional layer known as weakly supervised sensitive heat map (WSSH) was added to detect the ROI of the lesion at a test accuracy of 98.65%, while comparing with Class Activation Map that involved weakly supervised technology achieved 0.954. The main purpose is to learn a representation that collect the central localization of discriminative features in a retina image. CNN-WSSH model is able to highlight decisive features in a single forward pass for getting the best detection of lesions.

## Introduction

The World Health Organization (WHO) considers Diabetic Retinopathy (DR) a high-priority disease as it is fatal and likely to result in complications. Regular dilated eye examinations were carried out for all patients with diabetes as per WHO recommendation. Such examinations can identify eye pathology at early stages to enable early intervention and reduce the risk of progression to sight loss^1^.

The main classification phases were reported to detect the severity of Diabetic Retinopathy. DR is a microvascular disease developing either as non-proliferative DR (NPDR) or as proliferative DR (PDR). The main variation between them is based on the presence of neo-vascularization. The retinal microvasculature is compromised, and its permeability becomes susceptible to microaneurysm^2^. NPDR is subdivided into classes: 0: No apparent retinopathy 1: Mild: small outpunching in the tiny blood vessels' appear in the retina. 2: Moderate: The disease's progression causes damage to the blood vessels that nourish the retina, resulting in swelling, sight distort, and lose their ability to transport blood. 3: Severe Numerous hemorrhages and microaneurysms: occur within 4 quadrants of the retina, the cotton wool spots appear in 2 or more quadrants and intraretinal microvascular abnormalities are present in at least 1 quadrant of the retina.

Soft exudates (SE) or Cotton Wool Spots (CWS) appear due to the arteriole occlusion. This debris accumulation has a different shape appears as woolly white lesions in the Retinal Nerve Fiber Layer (RNFL). On the other hand, hard exudates (HE) are vivid yellow or white-colored entities on the retina. These entities appear waxy with sharp edges against the background from blood vessels. Hard exudates are caused by blood leakage from veins, and exudates have circular shapes around vessels.

4: PDR is an advanced stage of DR. It happens when flimsy and fragile blood vessels grow peculiarly from the retina into the vitreous, which is considered the leading cause of blindness problem. PDR can be characterized by neovascularization on the retina and the posterior surface of the vitreous and can lead to retinal detachment^3,4^. These new blood vessel developments are abnormal and lead to blood leaking inside the retina. The Wisconsin Epidemiologic Study of Diabetic Retinopathy (WESDR)^5^ found that in PDR, the risk of sight loss dramatically increases with progression.

Microaneurysms (MAs) appears as a red color circle in small proportions. As a result, optometrists regarded it as the first sign of diabetic retinopathy. MAs exist in a variety of sizes and shapes, depending on the patient's age. while, hemorrhages HMs occur as brown patches at the ends of blood vessels and are made up of blood vessel breaks. During this stage, blood leaks are referred to as "dot-and-blot" hemorrhages.

as comparative, Hard exudates are more advanced than other retinal lesions such microaneurysms and hemorrhages (red lesions). It arises with well-defined borders in a variety of sites and is one of the leading causes of vision loss, particularly when it develops near or on the fovea.

In this paper, the pre-trained Convolutional Neural Network (CNN) was developed to a Multilayer convolutional Neural Network Model (MCNN). CNN and MCNN are a first-hand cooperative detection and localization layer that classifies the input image and gathers mapping features from image calculation by applying a weakly supervised sensitive heat map (WSSH). Our project has many contributions; first & foremost, the structure accomplishes a DR raw dataset matching procedure to guide the matching movement heat mapping near the exudate's regions. The consolidated network is a step-set localization procedure by computing the lesion's point in the validation phase with matching flow-data training as a shape before instructing the weakly supervised (WS) for APTOS localization. On the other hand, the accuracy on image classification for the whole network was increased by separating its computation in typical images and DR images.

This study focuses on gathering and collecting the best features that can learn the network and avoid the error of detection.

For that purpose, we apply preprocessing technique and remove optic disc OD that has same pixel values of Exudates lesions. However, the efficient model depends on several factors: preparing the dataset with augmentation, fine tuning the model, preprocessing and network structure.

The rest of this paper is organized as follows. In “Literature review” section , the former research related to the CNN, and DR predictions and localization are discussed. “Methodology” section applies the suggested methodology, which consists of a deep learning framework, an Efficient Net model: and weakly supervised localization techniques. "Supervised learning CNN Works” section illustrates and analyzes the dataset description, the experimental settings, and results. Finally, the conclusion summarizes the applications of the study’s findings.

## Literature review

Vision-2020 is a global initiative that advocates for 'the Right to Sight' and established a partnership between the World Health Organization (WHO) and the International Agency for the Prevention of Blindness (IAPB), which launched in 1999. The two aims of eliminating avoidable blindness and decrease visual impairment are a global public health problem. However, automatic predicting approaches are expected to be developed to improve the efficiency and reliability of the results of pathological examination. Previously, numerous models were applied to obtain the best classification with segmentation, even with localized techniques such as the DWCE algorithm. It proposed for segmentation of digitized mammograms and implementation along with edge detection and morphological feature classification^6^; nevertheless, it is still a complicated issue and a tedious task to examine, size of lesions, and the similarity between pixels that appear in hard exudates (HE) and the optic disk (OD).

In essence, the convolutional neural network is an efficient branch of the neural network representing the core of deep learning. The utility of CNN has been expanded from detection to localization and segmentation^7^. Moreover, it makes significant breakthroughs in computer vision. Improving representation ability is a crucial problem in designing the model structure for handling complex tasks such as attribute analysis. Typical image processing methods such as contrast enhancement^8^, histogram analysis^9^, edge detection^10^, and matched filtering^11^ have been applied to identify different types of DR lesions.

Gondal et al.^28^ proposed CNN network for DR classification problem. The second part of model has been modified to apply weakly supervised object localization (WSL). Heatmaps generated for localization with Class Activation Maps. However, the model solves the localization problem but still needs development to eliminates error prediction. We enable an optic disc removal algorithm that has an impact feedback to enhance the detection.

### CNN classification

Several algorithms were proposed to solve the classification problem, such as Decision Tree, Support Vector Machine, and Random Forest to detect bright and dark DR lesions. The region-level classification was the best technique compared with pixel-level, considering the shape and size of the lesion. Pires et al.^12^ investigated a data-driven method to extract powerful abstract representations and applied it to investigate the ability of transfer learning (TL) in the context of DR screening at a particular area on the image. The presented technique denotes the patient immediately from the retinal examination pixels, without preliminary feature extraction for the image or lesion edges detection from an initial basic configuration.

A deep learning-based solution was created to highlight various methods and develop a robust framework. Lama et al.^13^ pioneered a novel red lesion detection method based on a new set of shape features named Dynamic Shape Features. Although it is an automatic telemedicine scheme, it decreased the AUC to 0.899 on Messidor and used a Random Forest (RF) classifier to discriminate between lesions and non-lesions spots. Shanthi et al.^14^ modified the Alexnet architecture for classification problems on the Messidor dataset after preprocessing phase, including the green channel to provide enhanced optic nerves and other vital features of the image by initialing into RGB channels. The goal of Alexnet is to compute a better computational aptitude to tackle the difficulties more effectively than other networks, such as Le-Net, Conv-Net. The presented technique utilizes principal component analysis (PCA) to separate the optic disc from the fundus dataset.

### Automatic detection of DR

The main advantage of automatic detection is assisting ophthalmologists to detect, classify, localize, or segment the damage that DR causes and ensure diagnosis at any phase of progression. Thus, it is considered more efficient at detection. Many studies have been proposed for DR detecting. These studies' methods can be generally categorized as follows : Unsupervised Methods, Supervised Methods, and weakly supervised learning.

Many DR detection approaches for retinal images have been introduced in recent years. The most efficient models are related to deep learning techniques especially, the supervised method^15^. Supervised approaches make use of the training phase that is manually managed and segmented by ophthalmologists. Various supervised classifiers have been used for this purpose such as Artificial Neural Networks (ANN), and K-Nearest Neighbor (K-NN) classifier. The inclusion of contextual semantic environments enhanced the performance of the classifier. The Gaussian mixture model^16^ produces segmentation by classifying each image pixel as vessel or no vessel depending on the pixel feature vector.

Unsupervised methods^17^ apply the Support Vector Machines (SVM) model with a basic line detector to the green channel of the retinal image. It is created to evaluate the average grey pixel-level along the lines of fixed length passing through the target pixel at different orientations. Maninis et al.^18^ proposed a CNN model called Deep Retinal Image Understanding ( DRIU ) algorithm that specializes in a base network for segmenting blood vessels and optic discs detection as an image-to-image regression task.

Several blood vessel segmentation methods have achieved satisfying results. Moreover, the segmentation approaches applied to retinal images with enhancement methods result in higher accuracy, applying a threshold method to obtain the edge binary map of DR^19^. A multilayered threshold method was proposed to segment the retinal dataset's blood vessels to detect neovascularization. The new blood vessels improved by two-dimensional wavelets. Abnormal blood vessels are detected using a sliding window procedure. Also, segmentation of blood vessels from the retina images may improve the detection process of any small spots of lesions, leading to vision disorders^20,21^.

## Methodology

**Figure 1:**
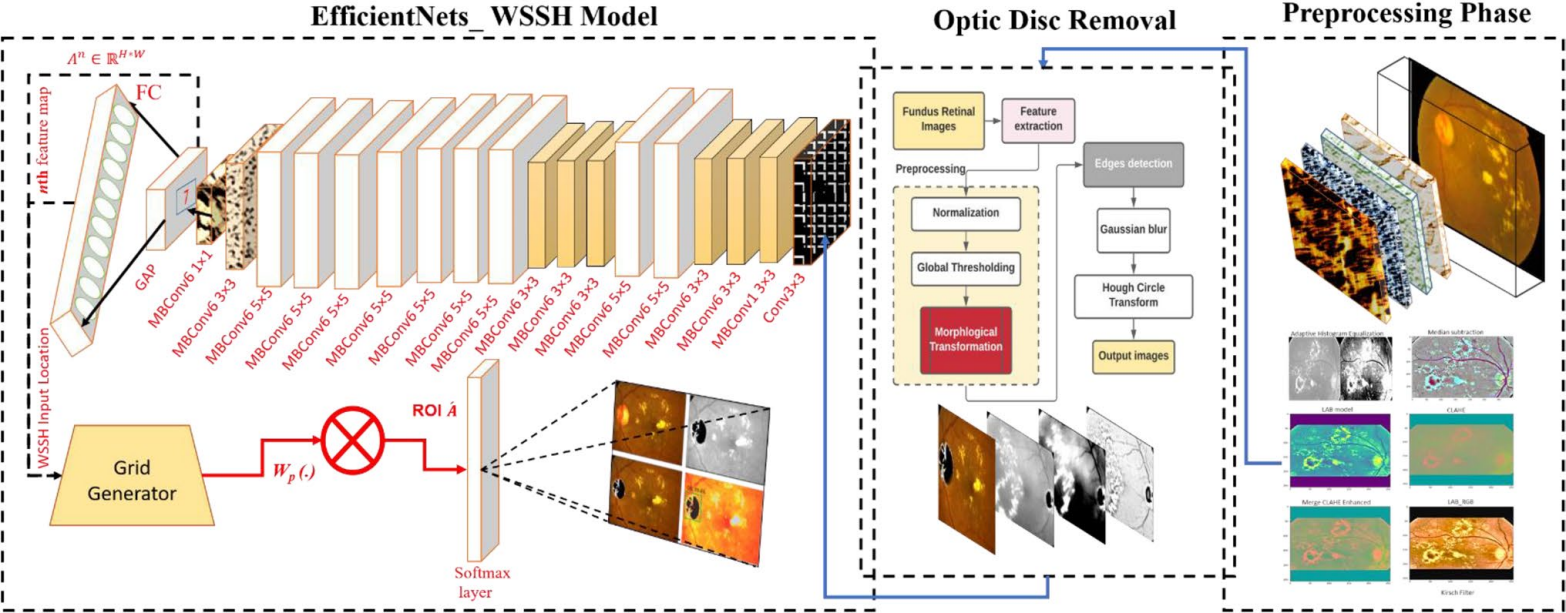
The schematic diagram of the proposed WSSH_DR model with multi-scale features strategy, modified preprocessing, ODR algorithm, and fully connected layer in EfficientNetB5 stage.

the proposed model to detect diabetic retinopathy consists of four main stages shown in Fig. 1. First and foremost, preprocessing methods are applied for preparing the data set and collect more features. Second, we used optic disk segment algorithm to cut the OD circles for eliminating any exudate's false prediction. After that, the model is fed through training phase for classifying each class label. Last, the layers are used to localize the impact of DR on the eye's patient.

### Pre-processing

Image preprocessing is an essential step to eliminate erroneous illumination contrast and noisy pixels with cumulative contrast between the background and the retinal blood vessels. The selection of preprocessing techniques affects the results accuracy on feature extraction and DR detection stages. Initially, the histogram of an image is used to produce a better image. The Gamma Correlation Eq. 1 is applied to mapping the relationship between a color value and its brightness. Then the adaptive histogram Eq. 2 is used to improve the image's contrast. The preprocessing algorithm steps are described in the algorithm (1).

1 $$I = 255*\left( \frac{I}{155} \right)^{\frac{1}{g}}$$

where *I* is the input value, *g* is Luminance intensity, 255 represent maximum gray level, but the adaptive histogram computed as:

2 $$P_{x} \left( i \right) = P\left( {x = i} \right) = \frac{{n_{i} }}{n},\quad 0 \le i \le L$$

where *x* is detached grayscale image, and *n*_*i*_ be the numeral of occurrences of gray level *i*, *L* being the total number of gray levels in the image, *n* being the total number of pixels of the image, and $P_{x} \left( i \right)$ being the image's histogram for pixel value *i*, that normalized to [0,1] as a black and white.


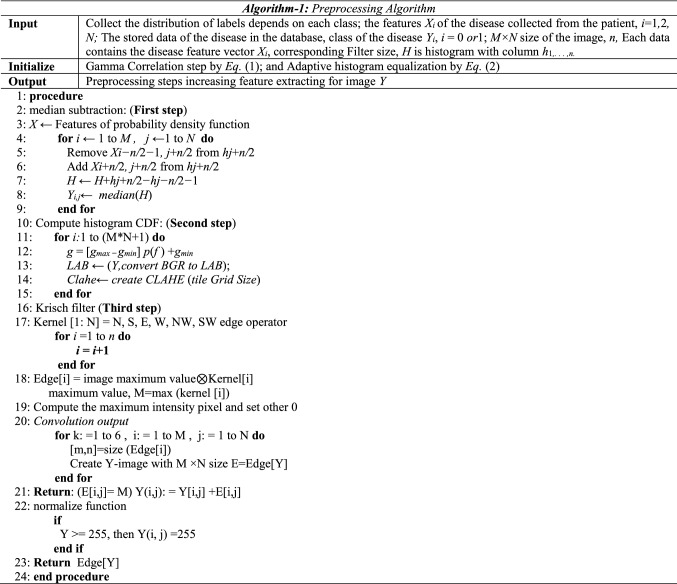


**First step:** Median filter is a creative technique applied to reduce or remove the impulsive noise and preserves edges of objects effectively, which does not require convolution. As described in algorithm-1, a median filter was applied to remove outliers from the image.

**Second step:**

- Converting image to LAB Color model
- Splitting the LAB image to different channels
- Applying contrast limited adaptive histogram equalization (CLAHE) to L-channel
- Merge the CLAHE enhanced L-channel with the *a* and *b* channel
- Converting images from the LAB Color model to the RGB model*.*

The Adaptive Histogram Equalization technology (AHE) is the technique of mapping every pixel in the image to an intensity value derived from the surrounded pixels by a transformation function. As computed by Eq. 2, the main advantage of Contrast Limited AHE (CLAHE) in comparison with AHE is that it prevents over-amplification of noise in the image’s homogeneous parts. Essentially, the CLAHE procedure^7^ improves the contrast among tiny regions of the lesion.

However, Contrast enhancement produces a better image than the original by changing the pixel intensities. The point processing adopted the pixel's values in the original input image to produce the values of the corresponding Eq. 3.

3 $$Y \left( {i, j} \right) = T \left[ {X\left( {i, j} \right)} \right]$$

where *X*(*i*,* j*) is the original raw image, *Y* (*i*,* j*) is the enhanced image, and *T* defines the transformation between the two images pixels in the enhanced image. Some of the point processing techniques includes: contrast stretching, global thresholding, and histogram equalization; particular mask processing methods contain averaging filters, sharpening filters, and local thresholding. Gamma correlation was applied after three processing techniques that mentioned in Algorithm 1. The main advantage of gamma correlation is clearing the images from darken range to lightening, which can collec the best features extracting for optic disk OD. In comparison, some images contain an optical disc that is difficult to detect and mysterious in its shape, so the data needs to be illuminated for getting the best edge region of OD.

**Figure 2:**
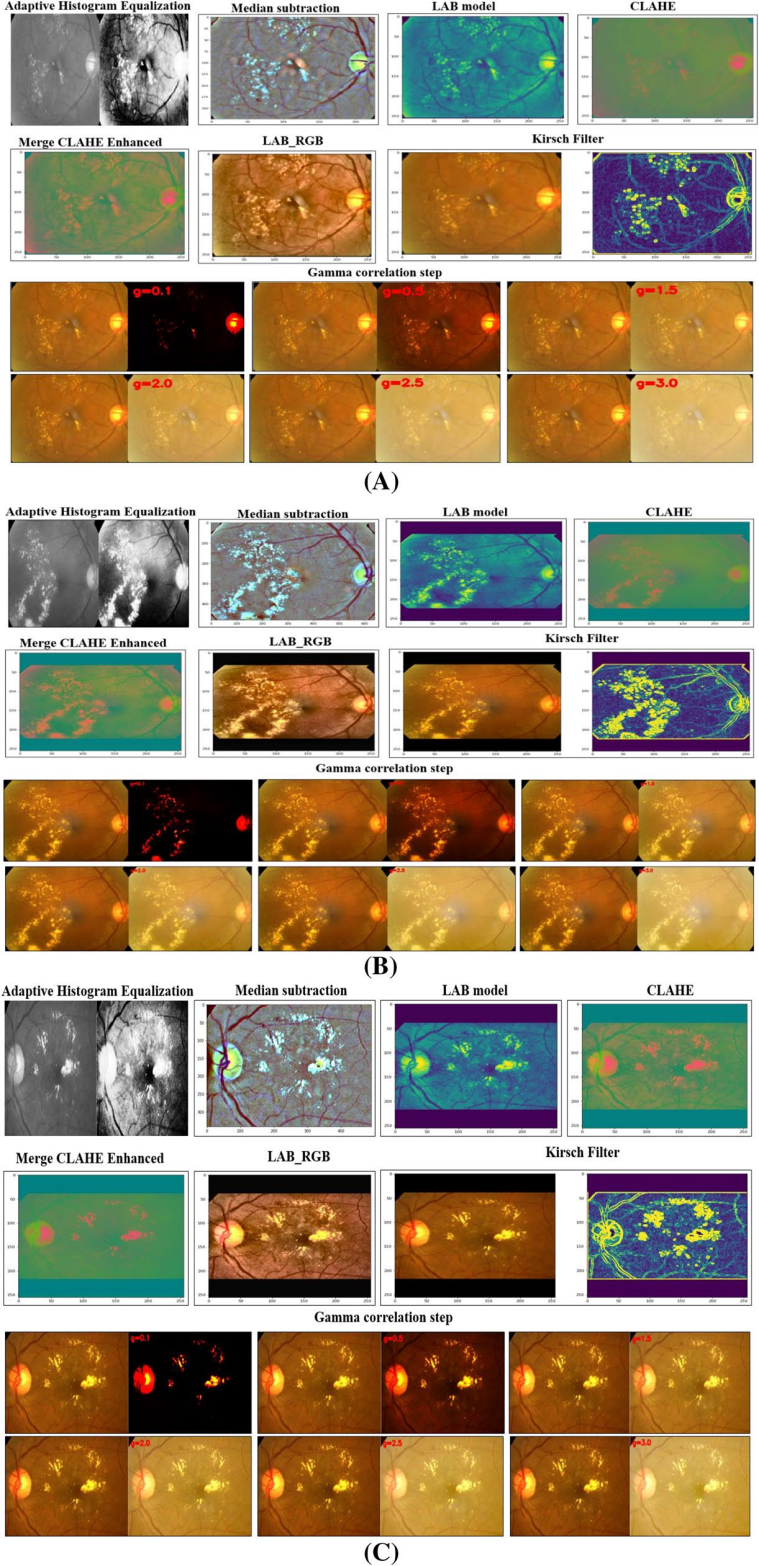
Preprocessing algorithm results (**A**–**C**).

**Third step:** Krisch filter is a non-linear edge detector that finds the maximum edge strength in a few predetermined directions. The main advantage of the kirsch filter is the efficiency of detecting maximum edges. The result analysis for this filter illustrates the last of preprocessing, and it used a convolution table as a 3 × 3 table of pixels. Figure 2 illustrates all the results of preprocessing algorithm-1.

## Optic disk removal

The optic disk removal algorithm's main advantage is producing the images without regions of interest (ROI) of optic disk (OD) pixels, thus increasing exudates detection performance as long as the exudates have a similar color of OD pixels, which appears yellow. The second advantage is solving the problem of false-positive detection. Following Eq. 4 will provide a mathematical background to normalize the grayscale image.

4 $$G\left( {i,j} \right) = L \left( {i, j} \right)L\left( {i,j + 1} \right)$$

L (i, j) is the grayscale of point (i, j), while images convert them to luminance components, and each pixel in grayscale has a value representing the shade of gray. The large images are resized into smaller images to avoid extensive time for training. The gradient and angle for the image are computed by Eq. 5.

5 $$G = \sqrt {G_{x}^{2} + G_{y}^{2} } ,\quad \theta = \tan^{ - 1} \left( {\frac{{G_{y} }}{{G_{x} }}} \right)$$

The pixels intensity for the first selected circle is computed, and then the optic disk is cut using specific algorithms^21,22^. The proposed Optic disk removal (ODR) algorithm-2 is enhanced, as shown in Fig. 1, by improving the contrast of the image components by (CLAHE) in the Global thresholding technique. Gaussian blur is added after edges detection to remove any false pixels. Horizontal direction *G*_*x*_ and vertical direction *G*_*y*_ are used for mask processing. The pixel value is extracted from the original pixel value of each image. It is a more expansive process than simple point processing, though it is robust. The grid mask for an input image will produce an output image of the same size as the input array. The features extracted from all images provide the same size to provide data consistency. The process used to determine OD's size when r represented the radius of OD is shown in Eq. 6. where a, b are the center coordinates.

6 $$r^{2} = \left( {x - a} \right)^{2} + \left( {y - b} \right)^{2}$$

7 $$x = a + r.\cos \left( \theta \right), \quad y = b + r.\sin \left( \theta \right)$$

The gradient *g*(x, y) is a threshold version of *f*(x, y) at some global threshold *T*. The global thresholding applied for the effective region outputs in a packed bit (0, 1) format described in Eq. (8). The Global thresholding is creative as the degree of intensity separation between the two peaks represents the image's background and object pixels.

8 $$g\left( {x,y} \right) = \left\{ {\begin{array}{*{20}c} 1 & {f\left( {x,y} \right) \ge T} \\ 0 & {Otherwise } \\ \end{array} } \right.$$

Two morphological operators are applied (Opening and Closing) obtained from the essential tasks of erosion and dilation for binary images. Opening operation Eq. 9, removes some of the small objects from the front pixels around the edges of ROI in the retinal image. Next operating, closing operator Eq. 10 removes small holes in the foreground by changing small islands of background into the foreground; also, it reduces noise in binary images. To reduce the size of foreground objects, we can erode pixels given several iterations.

9 $$Opening \,operation :A \circ B = \left( {A \ominus B} \right) \oplus B$$

10 $$Closing\, operation: A \bullet B = \left( {A \oplus B} \right) \ominus B$$

$\ominus$ and $\oplus$ denote erosion and dilation, respectively, A is binary images, and B is the structuring element. For grayscale morphology, the structuring functions (Denoting an image by *f*(x) and the structuring function by *b*(x), the grayscale dilation *f* by *b* is given by Eq. 11.

11 $$\left( {f \oplus b} \right)\left( x \right) = SUP_{y \in E} \left[ {f\left( y \right) + b\left( {x - y} \right)} \right]$$

**Figure 3:**
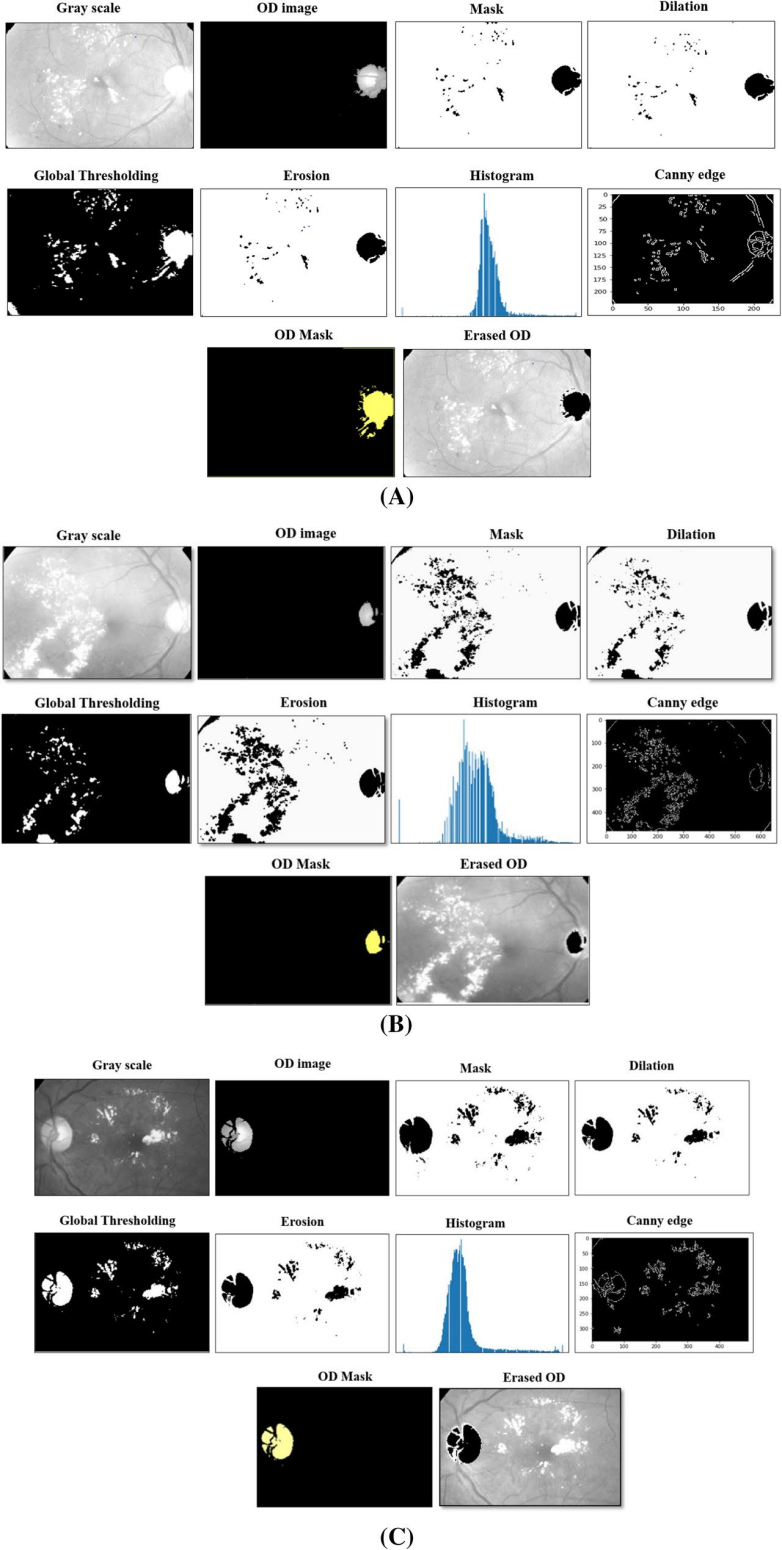
Optic disk removal detection (**A**–**C**).

The gamma compression function must first be removed via gamma expansion (linearization) to transform the image to a linear RGB color space. Finally, the Erosion operator removes pixels from edges to make an object smaller, while the dilation operator adds pixels around its edges to make an object larger. The threshold method appears at the neighbors of a pixel and changes its state if the number of differing neighbor pixels exceeds a threshold. Figure 3 demonstrates the processing result of removing the optic disk and describes all images step by step.

## Supervised learning *CNN Works*

Supervised learning model returns a function that predicts the correct result of *f*(x), measured by generalized performance on *Ƒ*_test_. It is generated by the learning algorithm using training data *F*_train_. The classification model is the first part of the study. The ability to separate the objects from ground truth and translate it as classes by using their attributes was considered the most crucial target of many studies. The performance of any algorithm classifier should be evaluated to demonstrate the error rate of the network. Generally, other criteria used to assess the model's performance include sensitivity for true positive rate and specificity for true negative rate.

### CNN for classification and localization (Algorithm 3)

Classification and localization procedure includes the following steps:

#### Initializing

The standard type of CNN is input sequential data *x* = [*x*_1_,*…..*, *x*_*T*_]*.* T is the length of the sequence and $x_{i} \in {\mathbb{R}}^{d}$ at each step. The convolution step described by the dot product between a filter vector $u \in {\mathbb{R}}^{md}$ and a concatenation vector representation *x*_*i:i*+*m−*1,_ which defines the operation as in Eq. 12.

12 $$c_{i} = \varphi \left( {u.x_{i:i + m - 1} + b} \right) where x_{i:i + m - 1} = x_{i} \parallel x_{i + 1} \parallel \ldots \parallel x_{i + m - 1}$$

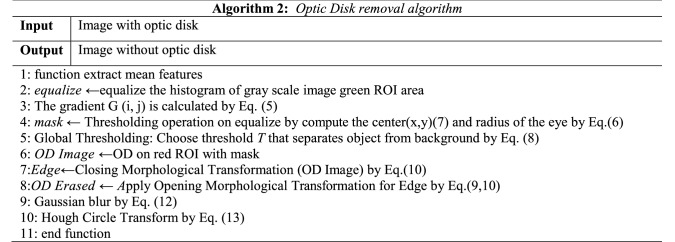
where (.) represents the dot product, *b* and $\varphi$ denote bias term and non-linear activation function, respectively. Additionally (||) is the concatenation operation for two vectors.

#### Update hyper-parameters

Its numbers of values derived via training the network have a significant effect on the model's predictive performance. It includes the number of layers, the kernel size and parameters that influence the gradient descent. The goal is to focus here that the parameters are governed and guide the learning method. Updating the equation for the weights involving the hyper-parameters described in Eq. 14:

13 $$CHT\left( {f\left( {c,r} \right)} \right)\mathop \smallint \limits_{\partial B,\left( c \right)}^{r} f\left( y \right)d\sigma \left( y \right){ }$$

14 $$\omega \leftarrow \omega^{\prime} = \omega - v\,\,where\,\,v \leftarrow v^{\prime} = \gamma v + \eta \frac{\partial C}{{\partial \omega }} + \frac{\lambda }{2n}\mathop \sum \limits_{i} \omega_{i}^{2}$$

where η is learning rate applied to determine the impact of each weight directly updated. Momentum γ ∈ (0, 1) described the inertia of the gradient update, means how much weight value is assigned to the last gradient. Weight decay λ ∈ (0, 1) is a weight regularization. The most crucial step is that the learning rate should decrease over time depending on decreasing epochs by a pre-defined factor or exponential decay.

#### EfficientNet model

We revisit the EfficientNet^23^ model, which defined the ConvNet layer *N* as a list of composed layers as Eq. 15. The goal is to develop the baseline in the model by leveraging a multi-objective neural design search that enhances the accuracy.

15 $$N = \odot_{i = 1}^{s} F_{i}^{{l_{i} }} \left( {X_{{\left( {H_{i} ,W_{i} ,C_{i} } \right)}} } \right)$$

However, scaling up the compound layers of our model increases the accuracy of detection. Where many deep layers will lead the model to be slower and without any improvement in detection. For that reason, we customized the layers of the model by fine-tuning and freezing others. On the contrary, the model prevents the vanishing gradient problem by preventing the weight from changing its value. Thus, it will completely stop the model from further training and produce poor results.

#### Normalization

- BN is an adaptive re-parameterization scheme and is used for solving the struggle of training deep models.
- Deep Neural Networks (DNN) will have the ability to optimize easily.
- The re-parameterization of any network can be effectively simple the problem of coordinating updates across all layers.

Mathematically, BN works to enhance the model overall by optimizing and updating the parameters. Let us define a mini-batch *B* of size *n*, when the normalization is applied to every activation independently. The specific activation *x*. Thus, the model has *n* values of this activation in the mini-batch, represented by *B* =  × 1…*n.* Normalize the batch of values *B* =  × 1…*n* using the mean and variance of the batch compute as:

16 $$\begin{aligned} & \mu_{B} = \left( \frac{a}{n} \right) \mathop \sum \limits_{i = 1}^{n} x_{i} \\ & \sigma_{B}^{2} = \left( \frac{1}{n} \right) \mathop \sum \limits_{i = 1}^{n} \left( {x_{i} - \mu_{B} } \right)^{2} \\ \end{aligned}$$

where, $\mu_{B}$ is the mini-batch mean, and $\sigma_{B}^{2}$ is the mini-batch variance.
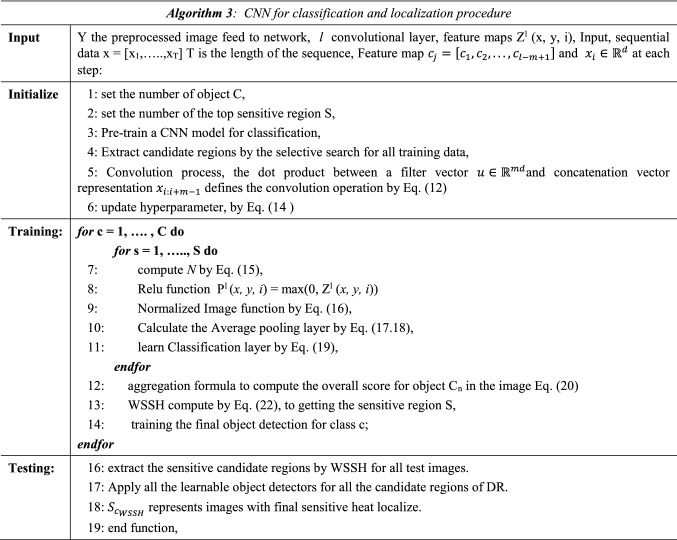


#### Global average pooling (GAP)

The proposed model's main problem is many parameters; consequently, the model requires a large amount of training data to avoid overfitting. Many different techniques succeeded in reducing the overfitting problem. One of them is the pooling process. The pooling process calculates the number of neurons in all convolutional layers and subsequently compares to the number of neurons stated.

It works to reduce the number of parameters drastically. Consequently, it reduces the training effort.

The Average pooling layer has more efficiency to increase overall the accuracy of the model. It computed by Eq. 17

17 $$Q^{l} \left( {x,y,i} \right) = P^{l} \left( {x,y,i} \right)\left( {\gamma + \alpha \mathop \sum \limits_{{j = M^{l} }} \left( {P^{l} \left( {x,y,i} \right)} \right)^{2} } \right)^{ - B}$$

where $Q^{l} \left( {x,y,i} \right)$ computes the response of the normalized activity from the ReLU with output $P_{{\left( {x,y,i} \right)}}^{l}$. This is achieved by multiplying the output with an inverse summation of squares plus an offset *γ* for all ReLU outputs within a layer *l.* The average pooling operator computes the mean response of each function channel obtained from the normalized output. It can be computed as Eq. 18

18 $$R^{l} \left( {x,y,i} \right) = \frac{{\mathop \sum \nolimits_{{x,y \in M_{{\left( {\overline{x},\overline{y},i} \right)}} }} Q^{l} \left( {x,y,i} \right)}}{{\left| {M_{{\left( {\overline{x},\overline{y},i} \right)}} } \right|}}$$

Summation weighted features were added in the latest convolutional layer of the model and adopted GAP layer performance to sum the average value of feature mapping. The average pooling operator computes the mean response of each feature channel obtained from the normalized output.

The GAP's drawback is that it is increasingly disposed to underestimating object size values because it considers all the activations. GAP loss stimulates the system for recognizing objects compared to global max pooling, which encourages recognizing one discriminative slice. The map calculates an average maximized value for whole parts discrimination to the interpreter of an object.

It should be noted that discriminative region localization is dependent on producing a bounding box process using a basic thresholding approach.

Furthermore, the Global average pooling (GAP) layer's purpose is to sum the average value of feature mapping related to the object's extent. the layer capable of calculating the average of a map and maximizing the value when the entire discriminative sections of the lesions are used.

#### Classification model

*Y* is fundus images with each image $A \in {\mathbb{R}}^{H*W}$ and the classification model *f* (.) that assigns to each image a class label *y* ∈ *c* where *c* ⊂ {*A*_0_,* A*_1_,* A*_2_,* A*_3_,* A*_4_}, *Y*_*S*_ = *F*(*A;ω*_*f*_*)* where *Y*_*s*_ is prediction and $\omega_{f}$ are the classification model parameters. The neural network learns the patterns from various images that should be included with detection. The most important part of model evaluation is the process of feature extracting. The network scans pixels of an image to recognize the object or the actual ground through this process. The main problem that affects adversely on the feature extraction is the noise included within images.

Thus, some preprocessing methods are added as mention in algorithm.1 on the Dataset to gather the highest values of features for learning our model. The probability of the classification step is computed using Eq. 19, where *w*_*k*_ is the corresponding set of GAP layer output. When *f*_*k*_ (*x*, *y*) define the activation of unit *k* in the final convolutional layer at (*x*, *y*).

19 $$I_{c2} \left( {x,y} \right) = \mathop \sum \limits_{k} \left( {\frac{{w_{k1}^{c} }}{4} + w_{k2}^{c} } \right)f_{k} \left( {x,y} \right) and P_{c} = \frac{{e^{{S_{c} }} }}{{\mathop \sum \nolimits_{c} e^{{S_{c} }} }}$$

S_c_ represents the summation of input and output of unit *c* at the final output layer of the fully connected network. $I_{c2} \left( {x,y} \right)$ is the importance ofiunit (*x*,* y*) at particular region. The class-sensitive heat saliency map is proposed to represent the values of *I*_*c*2_ (*x*, *y*), *I*_*c*3_(*x*,* y*), *I*_*c*4_(*x*,* y*), and *I*_*c*5_(*x*,* y*).

The forward and backward convolutional process describes the introduction of classifying each label of the input image as detailed in algorithm 4. 
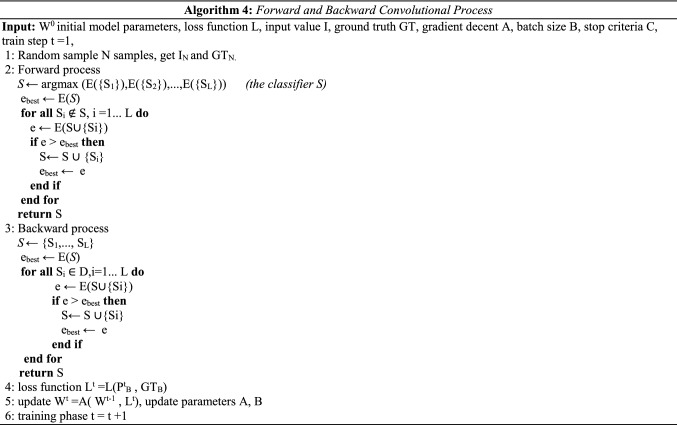


### Weakly supervised DR localization via WSSH

Generally, there are two approaches of determining localization, represented by bounding boxes or without bounding boxes labels. Weakly supervised localization (WSL) is still considered a challenging task that deals with image labels, splitting them into classes and creating the bounding boxes for all images. WSL concept used the automatically learning computing bounding boxes. The localization model is specified as *g* (.) that return *s* to the position *p* of the ROI, $$\mathrm{P} = g(A;\omega_{g} )$$$$\omega_{f}$$ the localization model parameters.$$\acute{A} \in R^{\acute{H}*\acute{W}}$$ Represent the ROI images obtained by $$\acute{A} = W_{P} \left( A \right)$$, *W* (.) is a warping operator. The main goal for a weakly supervised localization layer is avoiding bounding box annotations, while the transformation matrix W_p_ (.) was applied to arrange and extract the primary samples for the location of the (ROI) grid. After the classification process, Class Sensitive Heat Map (Weakly Supervised Sensitive HeatMap) is derivative based on the activations of the feature maps of the latest convolutional layer. The activation of the unit at spatial location (*x*,* y*) in the n*th* feature map is denoted as *f*_*n*_(*x*,* y*)*.* The class-sensitive heat map *H*_*c*_ for class c is defined by Eq. 20.

20 $$H_{c} \left( {x,y} \right) = \mathop \sum \limits_{n} w_{n}^{c} f_{n} \left( {x,y} \right)$$

$F\left( {x,y} \right) = \mathop \sum \limits_{n} w^{n} f^{n} \left( {x,y} \right)$ when $A_{n1} = \frac{1}{{N_{1} }}\mathop \sum \limits_{x,y = 1}^{n} f_{n} \left( {x,y} \right)$, where *N*_*1*_ represents the number of units in region *r*_*1*_. The pooling technique pools the (*n*th) feature map into units,$A_{n2} ,A_{n3} ,{ }A_{n4} ,{ }and{ }A_{n5}$. Then, all units will be fed into the softmax layer. The input of the softmax layer for category *c* is denoted as S_c_ and defines in Eq. 21.

21 $$S_{c} = \mathop \sum \limits_{n} w_{n1}^{c} A_{n1} + \mathop \sum \limits_{n} w_{n2}^{c} A_{n2}$$

where, *c* is the number of class, *S* is the score of probability, *n* feature map at $A^{n} \in {\mathbb{R}}^{H*W}$.

22 $$S_{{c\left( {WSSH} \right)}} = \frac{1}{{N_{1} }} \mathop \sum \limits_{x} \mathop \sum \limits_{y} \mathop \sum \limits_{n} W_{n}^{c} A_{x,y}^{n}$$

**Figure 4:**
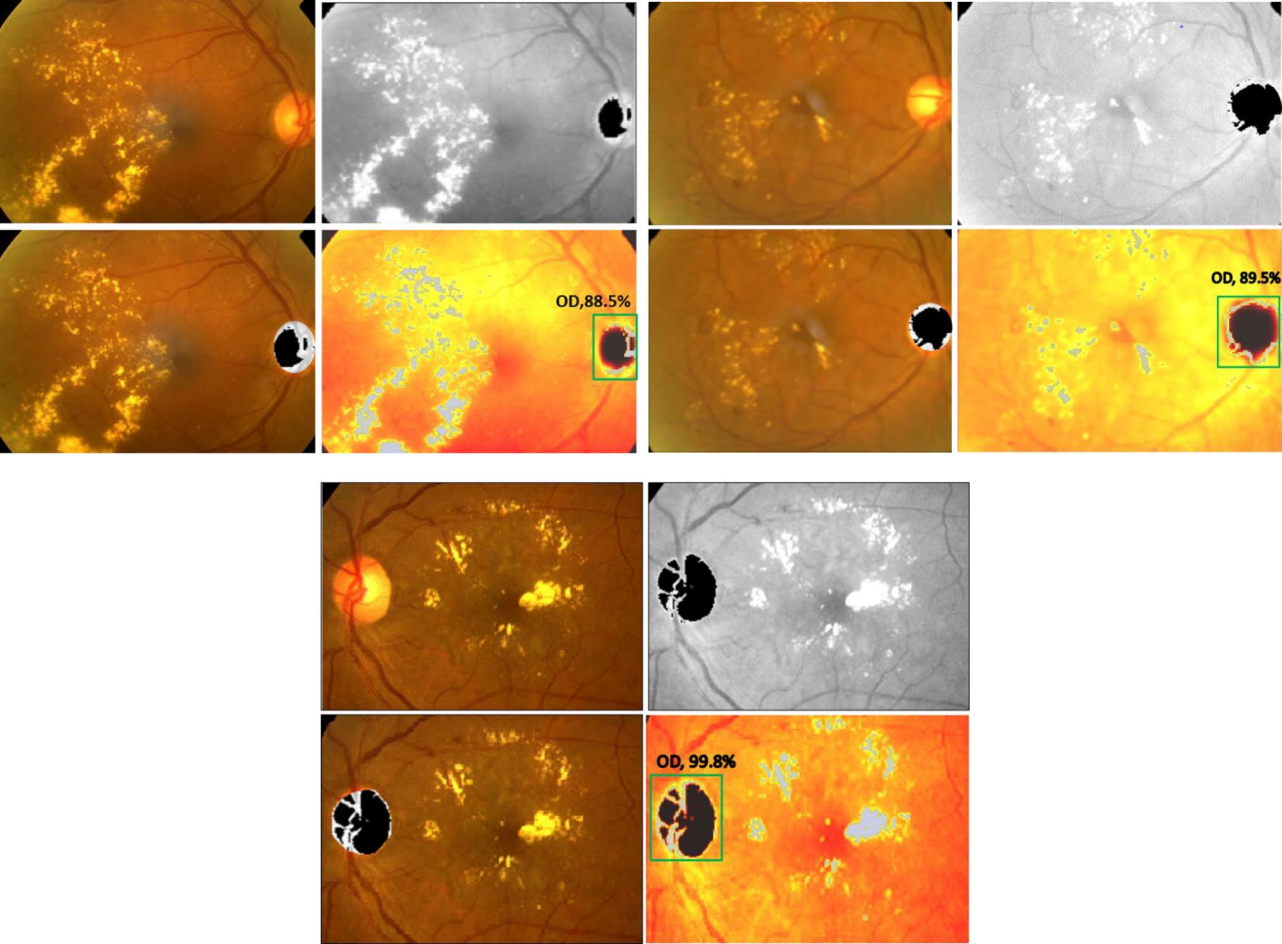
Weakly Supervised Sensitive Heat-map localization (WSSH).

$W_{n}^{c}$ is class feature weights, $A_{x,y}^{n}$ feature sensitive heat map. A neural architecture search is used to design a new baseline network, and scale it up to obtain high prediction performance, called by EfficientNets. This, perfectly solves the classification problem and faster than other models such as ResNet, DensNet, inception. Finally, revisiting the last layer of the model responsible for localizing the region of DR, Fig. 4 illustrates the Weakly Supervised Sensitive Heatmap for lesion localization without OD.

## Results and evaluation

**Figure 5:**
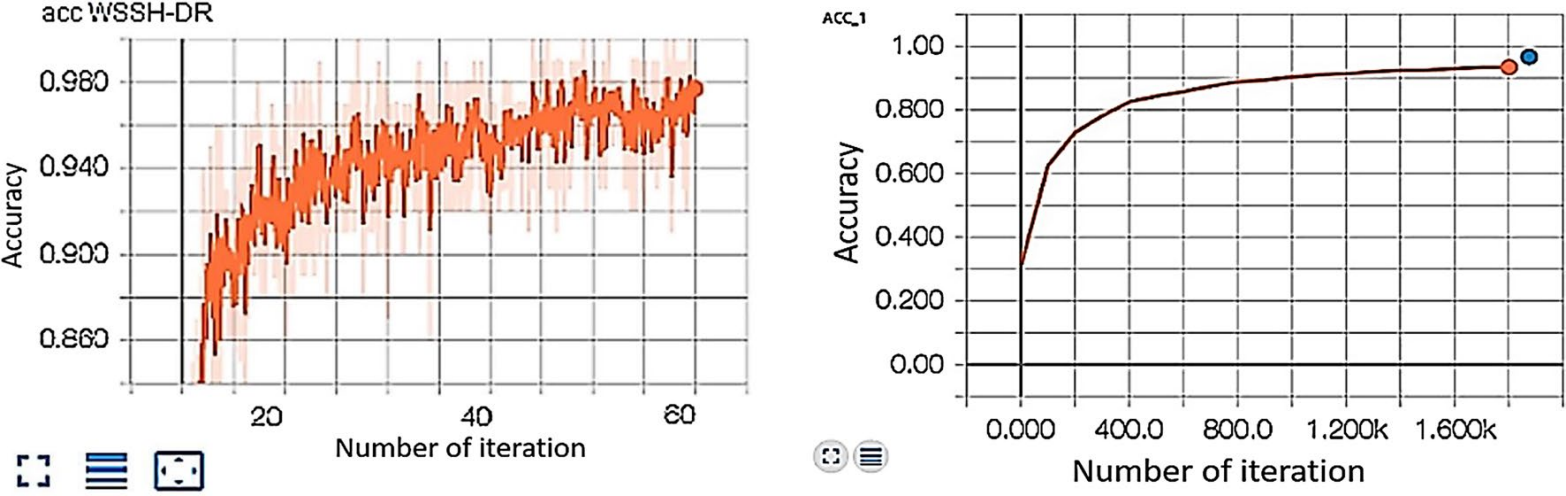
Accuracy of detection for WSSH-DR.

**Figure 6:**
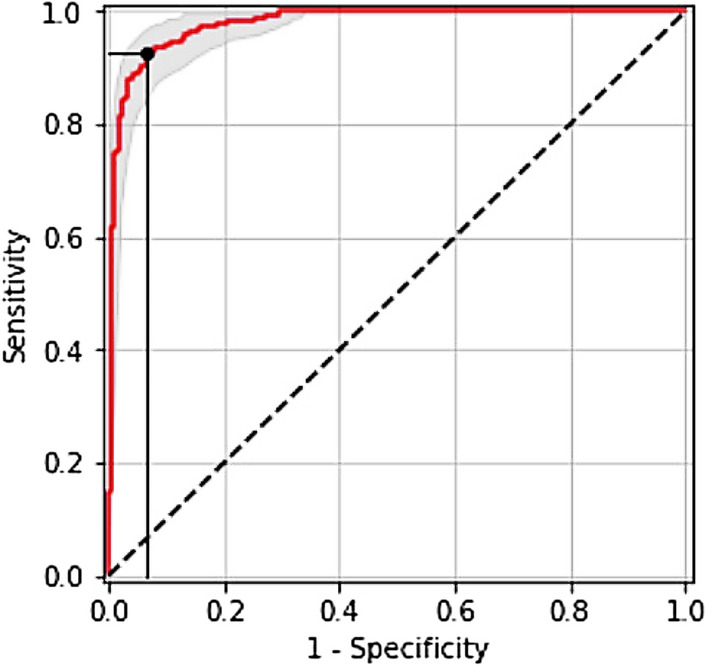
WSSH ROC using sensitivity and specificity metrics.

**Figure 7:**
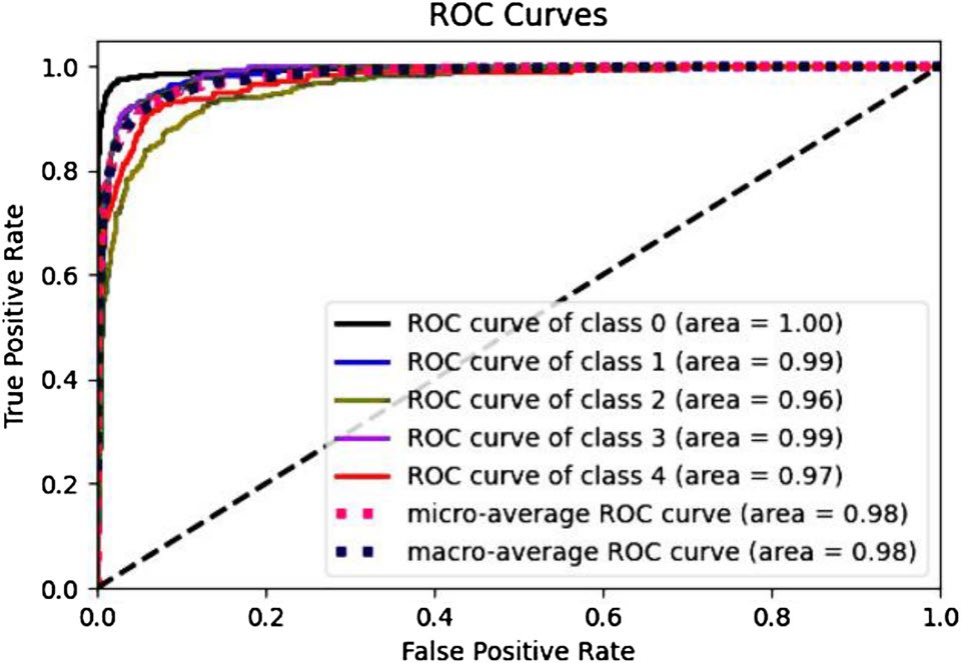
Receiver Operating Characteristic (ROC) curves for each class of DR lesions.

The parameter initialization methods are set to train the whole network by using the momentum optimizer, while *MESSIDOR*^31^ dataset was described and published in 2008. However, it contains four different classes with variable intrinsic and extrinsic features. The network trained a total of 60 epochs with an initial weight decay of 0.0001 for learning rate. The learning rate sets at 0.1 at epoch 25, then 0.01 at epoch 37, then 0.001 at epoch 45, and 0.0001 to epoch 60, while the batch normalization is applied after the convolution layer and the non-linearity layer. Figure 5 shows the accuracy of detection for our CNN-WSSH model for classification and localization of the lesions of DR. Table 1 compares the performance metrics for the test phase with different DR classes. Predict the performance sensitivity and specificity metrics of the network illustrated in Fig 6. Figure 7 illustrated Receiver Operating Characteristic (ROC) curves for each class of DR lesions of WSSH which compared with Rao^32^ as describing in Table 2.

**Table 1:** Performance metrics for test phase with different DR classes.

Input class	WSSH AUC	Rao^32^ AUC
DR A0 (healthy retina)	**1.00**	0.99
DR A1 MILD NPDR	**0.99**	0.97
DR A 2 MODERATE NPDR	**0.96**	0.94
DR A3 SEVERE NPDR	**0.99**	0.97
DR A4 PDR	**0.97**	0.96

The bold indicate to our results that used to comparative with other studies.

**Table 2:** ROC comparative results.

Model	Optimizer	Micro Average AUC
VGG19^32^	Stochastic Gradient Descent	0.6288
VGG16^32^	Adam	0.7037
GoogLeNet^38^	–	0.81
InceptionResNetV2^32^	Adam	0.97
EfficientNetB5 (ours)	Momentum	**0.98**

The bold indicate to our results that used to comparative with other studies.

Table 3 emphasizes the overall accuracy of the three models used, EfficientNetB5, Densenet201, and ResNet50. In comparison to other methods at different techniques.

**Table 3:** Level performance comparison with different techniques for Lesion detection.

Authors	Method	AUC
Kumar et al.^24^	ADDR classified DR principal	94.44% 87.5%
Bhatia et al.^25^	SVM PNN	90.76% 87.69%
García et al.^26^	SVM	82
Haar et al.^27^	_	72
Gondal et al.^28^	CNN-CAM	0.954
Shanthi et al.^14^	Modified Alexnet	95.6%
Dash et al.^29^	Star networked pixel tracking 2-D Gabor wavelet to SVM	95.83% 94.69% 93.2%
Verma et al.^20^	Random Forests technique (normal cases) (moderate and severe)	90% 87.5%
Niemeijer et al.^30^	-	95
EfficientNetB5 (ours)	CNN-WSSH	**98.4%**
Densenet201 (ours)	CNN-Classification	**94.5%**
ResNet50 (ours)	CNN-Classification	**89.7%**

However, It should be noted that discriminative region localization is dependent on producing a bounding box process using a basic thresholding approach.

Furthermore, the Global average pooling (GAP) layer's purpose is to sum the average value of feature mapping related to the object's extent. the layer capable of calculating the average of a map and maximizing the value when the entire discriminative sections of the lesions are used.

The statistical performance of WSSH model computed by ROC curve by plotting the true positive rate (TPR) against the false positive rate (FPR).

Figure 7 describes the analysis result that provides each class label of the dataset to select possibly optimal average ROC curve.

Momentum Optimizer is an addition to the gradient descent (GD) method that is used in search to develop inertia in a direction in the search space, overcome oscillations of noisy gradients, and coast through flat regions in the search space. Table 2 shows the impact of implementation to our model by achieves higher accuracy than comparing with other optimization algorithms. The best value of.

Momentum optimization was 0.9. However, an image is considered to have exudates if at least one candidate is classified as an.

exudate.

Figure 6 describes the comparative result between sensitivity and specificity metrics of our model when number of images is 1200. While Table 4 summarizes the results of two operating points in the ROC curves were high sensitivity and high specificity are obtained. Also its compressions result with other models.

**Table 4:** Comparison of sensitivity and specificity values for the exudates classification.

Authors	No. of Images	Sensitivity	Specificity
Walter et al.^33^	30	92.8	–
Osareh et al.^34^	300	96	94.6
Gardner et al.^35^	58	88.4	83.5
Hatanaka et al.^36^	109	77	83
Kumar et al.^9^	158	88.45	95.5
SujithKumar et al.^24^	–	94.44	87.5
Zhang et al.^37^	30	90.6	–
Proposed method (WSSH)	1200	**98.2**	**96.4**

The bold indicate to our results that used to comparative with other studies.

## Conclusion

Early diagnosis of subclinical DR will offer appropriate recognition and control for patients at a greater DR progression danger. Currently, strict regulation of blood glucose levels and other changes in risk factors are the only prevention methods recommended at early stages of DR. The ideal technique is feature extraction by using some preprocessing algorithm for increasing feature learning that feeds the network. In contrast, an optic disk removal algorithm is added to cut the optic disk circular because it results in a false-positive results with Exudates and OD appears at the same pixel color as Exudates. A localizing part detects spots of exudates clearly and avoids false learning errors during training. All preprocessing steps with preparing conspicuous data and removing optic disc have significantly impacted the accuracy and avoided many errors in the learning phase. It can be concluded that any model’s detection depends on the data used in the learning stage. Therefore, if the network feeds with the dark, tenebrous, dim Dataset, it will decrease the model's efficiency.
